# TLR9 signaling requires ligand-induced phosphorylation of two specific tyrosine residues by EGFR and Syk

**DOI:** 10.1128/mbio.00276-25

**Published:** 2025-09-22

**Authors:** Manoj Veleeparambil, Chenyao Wang, Patricia M. Kessler, Pracheta Sengupta, Santanu Das, Ritu Chakravarti, Belinda Willard, Ganes C. Sen, Saurabh Chattopadhyay

**Affiliations:** 1 Department of Microbiology, Immunology and Molecular Genetics, University of Kentucky College of Medicine 12252, Lexington, Kentucky, USA; 2 Department of Immunity and Inflammation, Lerner Research Institute, Cleveland Clinic 2569, Cleveland, Ohio, USA; Stony Brook University, Stony Brook, New York, USA

**Keywords:** TLR9, innate imunity, Syk, EGFR, cell signaling, toll-like receptors, Lyn, tyrosine phosphorylation

## Abstract

Toll-like receptors (TLRs) are transmembrane proteins that recognize microbial components or cellular danger signals and activate intracellular signaling pathways, leading to induction of anti-microbial and inflammatory genes. Inactive TLRs require ligand-induced activation to recruit adaptor proteins, e.g., MyD88, to trigger the synthesis of cytokines and interferons. TLR9 is an endosomal membrane-bound protein that binds to CpG-containing microbial DNA or endogenous signals from dead cells or tissue damage. We showed that TLR9 activation requires EGFR, a tyrosine (Tyr) kinase, which interacts with and phosphorylates the cytoplasmic domain of TLR9. Blocking EGFR activity pharmacologically, or knocking out EGFR gene in myeloid cells, suppressed lethal TLR9-induced hepatotoxicity. Here, we reveal that TLR9 required two Src family of kinases, Syk and Lyn, which, together with EGFR, led to phosphorylation and activation of TLR9. Lack of either of these kinases inhibited TLR9-MyD88 interaction, thereby inhibiting TLR9-mediated gene induction. Unlike EGFR, which constitutively binds TLR9, activated Syk interacted with TLR9 in a CpG-dependent manner. Activated Syk interacted with TLR9 and was critical for activating TLR9-bound EGFR. Quantitative mass spectrometric analyses revealed that TLR9 was phosphorylated sequentially on Tyr^870^ and Tyr^980^ by Syk and EGFR, respectively. Mutation of either of these tyrosines led to complete loss of TLR9-induced cytokine production. For activation, Syk was phosphorylated by Lyn, which was activated by CpG-mediated scavenger-receptor A, and surprisingly, independent of TLR9. In summary, our results uncovered the molecular details of TLR9 activation by its Tyr-phosphorylation, which is critical for TLR9-mediated intracellular signaling.

**IMPORTANCE:**

Toll-like receptors (TLRs) are critical components of cellular innate immune responses to microbial infection or tissue damage. TLRs are transmembrane proteins that require activation to mount a successful host response; TLR mutations are associated with human diseases. TLR ligands are also used as vaccine adjuvants to amplify the inflammatory responses of the host, activation of TLRs, and their regulation are essential. Here, we report the molecular mechanisms of TLR9 activation by tyrosine phosphorylation of its cytoplasmic domain. TLR9 interacts with EGFR and Syk, the tyrosine kinases, which phosphorylate two specific tyrosine residues on the TLR9 cytoplasmic domain. Mutation of these tyrosine residues or deficiency of these tyrosine kinases leads to impaired TLR9 signaling. Therefore, our results elucidate the early events of TLR9 signaling with implications in inflammatory diseases.

## INTRODUCTION

Cellular pattern recognition receptors (PRRs), e.g., Toll-like receptors (TLRs), RIG-I-like receptors (RLRs), cyclic GMP-AMP synthase (cGAS) sense viral nucleic acids, also known as pathogen-associated molecular patterns (PAMPs) (1, 2). PRRs also detect cellular danger signals, known as damage-associated molecular patterns (DAMPs), generated from damaged cells and tissues (3, 4). TLRs are transmembrane proteins expressed either on the plasma membrane or endosomal membrane, whereas RLRs and cGAS are localized in the cytosol (5). Specific locations of the PRRs enable them to detect PAMPs from different families of viruses. Upon sensing PAMPs or DAMPs, the PRRs get activated and signal via intracellular adaptor proteins, e.g., TRIF, MyD88, MAVS, and STING, to activate Ser/Thr kinases, e.g., TBK1 and IKKε. Activation of these kinases is required for phosphorylating the inactive transcription factors, such as interferon regulatory factor 3 (IRF3) (6). Phosphorylation of IRF3 causes it to dimerize and translocate into the nucleus, leading to the transcriptionally active IRF3, which binds the specific gene promoters to induce antiviral genes, e.g., interferons (IFNs) and IFN-stimulated genes (ISGs). Many of these newly induced ISGs act as viral restriction factors by interfering with specific stages of the viral life cycle to inhibit viral replication (7, 8). PRRs activate, in addition to IRF3, NF-κB, a pro-inflammatory transcription factor, which is responsible for inflammatory gene expression (2, 9–11). Cooperative action of IRF3 and NF-κB is essential for the synthesis of IFNβ, an antiviral cytokine. IFNβ, after being synthesized in the virus-infected cells, gets secreted and acts on infected or neighboring uninfected cells to amplify the expression of antiviral genes (12, 13). Therefore, PRR activation is essential for the successful outcomes of the viral infection and inflammatory responses of the host.

TLR9, a member of the TLR family, expressed primarily in immune cells, e.g., dendritic cells, macrophages, and B cells, is an endosomal membrane-bound TLR. TLR9 detects the unmethylated CpG containing DNA from PAMPs and DAMPs. Recent studies indicated that TLR9 can also recognize mitochondrial DNA (mtDNA), released from diseased tissues or damaged cells (14, 15). Inactive TLR9 remains as a monomer, which, upon binding CpG DNA by its ectodomain in the endosomal lumen, dimerizes and changes its conformation. The conformational change allows the cytoplasmic domain of TLR9 to recruit its adaptor protein, MyD88. TLR9/MyD88 complex triggers a cascade of intracellular signaling pathways via TRAF6 and IKK to activate the NF-κB. Activation of NF-κB leads to the induction of numerous pro-inflammatory genes, which are essential for regulating cellular inflammatory responses. TLR9 also activates IRF7 via phosphorylation by IRAK1 to induce type-I IFNs (16, 17). UNC93B1, a cofactor for other endosomal TLRs, is involved in TLR9 processing, which is critical for its localization and downstream signaling (18–20). The physiological relevance of TLR9 functions has been established in both viral and bacterial infection models. In addition, the TLR9 ligand, CpG, used as an adjuvant for vaccines, has implications in B cell functions. A plethora of studies have connected TLR9 with non-microbial diseases, e.g., hepatitis and autoimmune diseases (21–23). In such scenarios, TLR9 activation is caused by mtDNA released from dead cells.

TLRs require post-translational modifications, e.g., Tyr phosphorylation of their cytoplasmic domains, for triggering intracellular signaling pathways (24–27). We showed, using extensive biochemical and genetic approaches, the role of TLR3 Tyr phosphorylation in its antiviral and pro-inflammatory functions (27–29). Phosphorylation of TLR3 is critical for recruiting its adaptor protein, TRIF. EGFR and Src phosphorylate two Tyr residues of TLR3 cytoplasmic domain; mutation of either of these two residues results in impaired TLR3 signaling (29). We expanded the role of EGFR in TLR9 phosphorylation and intracellular signaling. EGFR remains bound constitutively to TLR9, and ligation with CpG leads to EGFR-mediated phosphorylation of TLR9 (30). Phosphorylated TLR9 is critical for interaction with its adaptor protein, MyD88, and activating downstream signaling. EGFR inhibitors, often used clinically to treat cancer, block TLR9 signaling *in vitro* and protect mice from TLR9-induced hepatotoxicity. The role of EGFR-mediated Tyr phosphorylation is known in TLR9 functions (30). However, the molecular details of TLR9 activation leading to its Tyr phosphorylation are incompletely understood. In the current study, we revealed the stepwise activation mechanism of TLR9 by EGFR and Syk-mediated phosphorylation of two Tyr residues in the TLR9 cytoplasmic domain. We used a combination of biochemical, genetic, and proteomic approaches to uncover the early events of TLR9 signaling that shapes the eventual outcome of TLR9-mediated cellular responses.

## RESULTS

### Lyn and Syk, in addition to EGFR, are required for TLR9-MyD88 interaction and TLR9-mediated gene induction

**Fig 1:**
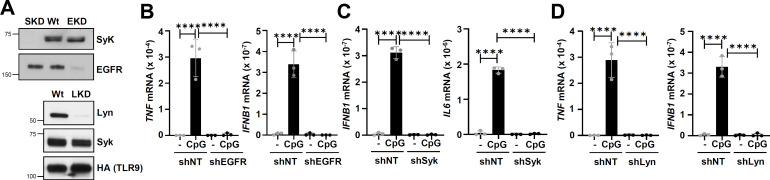
EGFR, Lyn, and Syk are required for TLR9-induced genes. (**A**) EGFR, Syk, and Lyn expression levels in shNT (Wt) and different gene knockdown 293XL-hTLR9-HA cells were analyzed by immunoblot; shRNA targeting Syk (SKD), shRNA targeting EGFR (EKD), and shRNA targeting Lyn (LKD). (**B**) 293XL-hTLR9-HA cells expressing non-targeting shRNA control (shNT) and shRNA targeting EGFR (shEGFR) were treated with CpG ODN (10 µg/mL) for 6 h, and the induced *TNF* and *IFNB1* mRNAs were measured by qRT-PCR. (**C**) 293XL-hTLR9-HA expressing shRNA targeting Syk (shSyk) and shNT were treated as in “B,” and *IFNB1* and *IL6* mRNAs were measured by qRT-PCR. (**D**) ShNT and 293XL-hTLR9-HA expressing shRNA targeting Lyn (shLyn) were used to measure *TNF* and *IFNB1* mRNA induction by qRT-PCR. The data represent mean ± SD (**B–D**), ****P < 0.0001.

**Fig 2:**
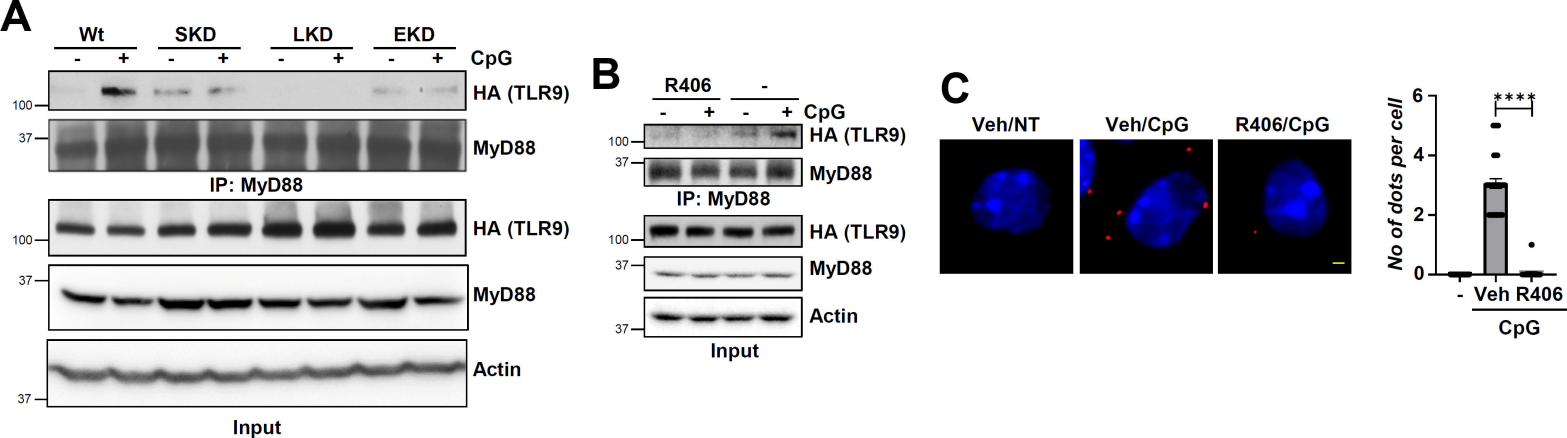
Ligand-induced MyD88 recruitment by TLR9 requires EGFR, Lyn, and Syk. (**A**) Wt, shSyk (SKD), shLyn (LKD), and shEGFR (EKD) 293XL-hTLR9-HA cells were treated with CpG ODN for 30 min. Cell extracts were immunoprecipitated with anti-MyD88 followed by immunoblot with anti-MyD88 and anti-HA for TLR9. (**B**) 293XL-hTLR9-HA cells were pretreated with DMSO (-) or the Syk kinase inhibitor (R406) (10 µM) for 1 h and then for 30 min CpG DNA along with DMSO or R406. Cell extracts were immunoprecipitated with anti-MyD88 followed by immunoblot with anti-MyD88 and anti-HA for TLR9. (**C**) RAW264.7 cells were treated with CpG in the absence or the presence of R406. The cells were then immunostained using anti-TLR9 and anti-Myd88 antibodies and analyzed by proximity ligation assay; scale bar—1 µm. Veh, DMSO. At least 35 cells were quantified from each independent experiment. The data represent mean ± SD, ****P < 0.0001.

We showed that EGFR activity is essential for TLR9-mediated gene induction (30). To evaluate the role of Src family kinases (SFKs), particularly Lyn and Syk, in TLR9-induced genes, we generated knockdown (KD) HEK293XL (HEK)-expressing TLR9 (HEK-TLR9) cell lines (EKD: EGFR KD, SKD: Syk KD, and LKD: Lyn KD) by stably expressing kinase-specific shRNAs. Expression of specific shRNA, as expected, did not alter the expression of other Tyr kinases or HA-TLR9 (Fig. 1A). Using these cells, we evaluated TLR9-induced gene expression. EKD cells, as expected, did not induce TNF or IFNB1 upon CpG treatment compared to the control cells, expressing non-targeting (NT) shRNA (Fig. 1B). Both SKD and LKD cells, like EKD cells, also failed to induce TLR9-mediated genes (TNF, IL6, and IFNB1) upon CpG treatment (Fig. 1C and D), indicating functional requirements of Lyn and Syk in TLR9-mediated gene induction. TLR9 interacts with its adaptor, MyD88, in a CpG-dependent manner for gene induction, and EGFR is essential for TLR9-MyD88 complex formation (30). We evaluated whether Lyn and Syk were required for TLR9-MyD88 interaction. Indeed, co-immunoprecipitation (co-IP) results indicated TLR9-MyD88 interaction was inhibited in SKD, LKD cells, and, expectedly, in EKD cells (Fig. 2A), suggesting these Tyr kinases were essential for TLR9-MyD88 complex formation. We validated these results using R406, a Syk kinase inhibitor, which, as expected, also inhibited TLR9-MyD88 interaction (Fig. 2B). We further confirmed these results using proximity ligation assay (PLA), a sensitive method that detects two closely located proteins in RAW264.7 mouse macrophages. CpG ODN treatment caused interaction of TLR9 and MyD88 (Fig. 2C, middle panel, red dots), which was strongly inhibited by R406 (Fig. 2C, right panel). Together, EGFR, Lyn, and Syk were required for TLR9-MyD88 interaction and, subsequently, TLR9-mediated gene induction.

### TLR9 interacts with activated Syk

**Fig 3:**
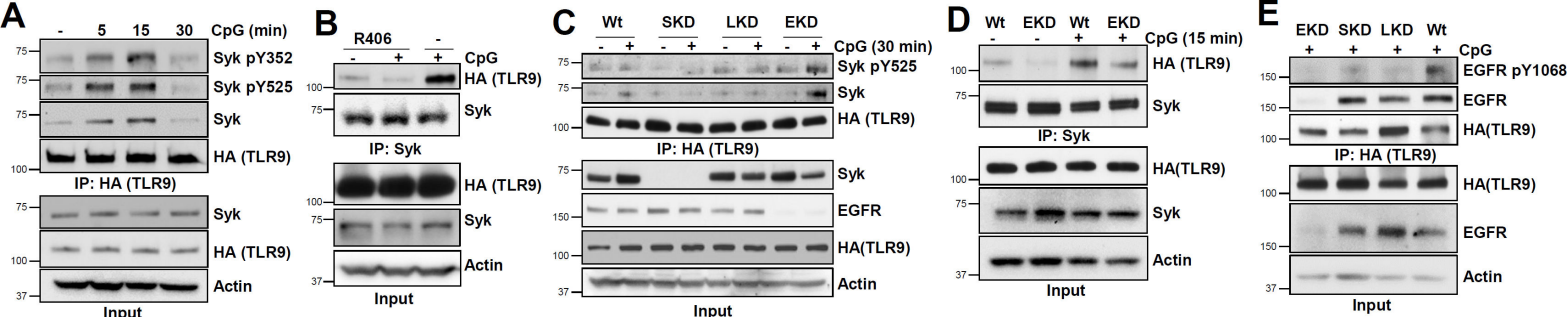
Activated Syk interacts with TLR9 transiently, EGFR inhibits the stability of the TLR9-Syk complex. (**A**) 293XL-hTLR9-HA cells were treated with CpG ODN for the indicated time, and cell extracts were immunoprecipitated with anti-HA for TLR9 and immunoblotted with the indicated antibodies. (**B**) 293XL-hTLR9-HA cells were pretreated with DMSO (-) or the Syk inhibitor (R406) (10 µM) for 1 h and then treated with CpG ODN along with DMSO or R406 for 15 min. Cell extracts were immunoprecipitated with anti-Syk and immunoblotted with anti-HA for TLR9. (**C**) ShNT (Wt), shSyk (SKD), shLyn (LKD), and shEGFR (EKD) 293XL-hTLR9-HA cells were treated with CpG ODN for 30 min, and the cell extracts were immunoprecipitated with anti-HA for TLR9 and immunoblotted with the indicated antibodies. (**D**) 293XL-hTLR9-HA shNT cells (Wt) and EKD cells were treated with CpG ODN for 15 min, cell extracts were immunoprecipitated with anti-Syk and immunoblotted with anti-HA for TLR9 and anti-Syk. (**E**) Cells and treatment as in “**D**,” cell extracts were immunoprecipitated with anti-HA for TLR9 and immunoblotted with the indicated antibodies.

To investigate whether Syk gets recruited to the TLR9 signaling complex, we performed co-IP studies. CpG stimulation caused rapid recruitment of phosphorylated Syk (pSyk) to TLR9 (Fig. 3A). R406 treatment reduced Syk interaction with TLR9 (Fig. 3B), indicating that activation of Syk was required for TLR9 interaction. TLR9-Syk complex was transient; Syk was recruited to TLR9 in the early phase (5 and 15 min) but dissociated from TLR9 30 min post-CpG treatment (Fig. 3A). TLR9-Syk complex, however, was stabilized in EKD cells, compared with Wt, LKD, or SKD cells 30-min post-CpG treatment (Fig. 3C), suggesting that dissociation of Syk from TLR9 was EGFR-dependent. EGFR, however, was not required for recruitment of Syk to TLR9; EKD cells also formed TLR9-Syk complex upon CpG treatment (Fig. 3D). Syk and Lyn, on the other hand, did not affect TLR9-EGFR binding; in CpG-treated cells, TLR9-EGFR interaction was unaffected in SKD and LKD cells (Fig. 3E). TLR9-bound EGFR was active, analyzed by phosphorylation of its cytoplasmic domain (Y^1068^), only in Wt but not in SKD or LKD cells (Fig. 3E), indicating that Syk and Lyn, although not required for TLR9-EGFR interaction, were required for activation of TLR9-bound EGFR. Therefore, EGFR regulated TLR9-Syk interaction; however, although Syk and Lyn had no role in TLR9-EGFR interaction, they were required to activate TLR9-bound EGFR.

### The cytoplasmic domain of endosomal EGFR was sufficient for TLR9 signaling

**Fig 4:**
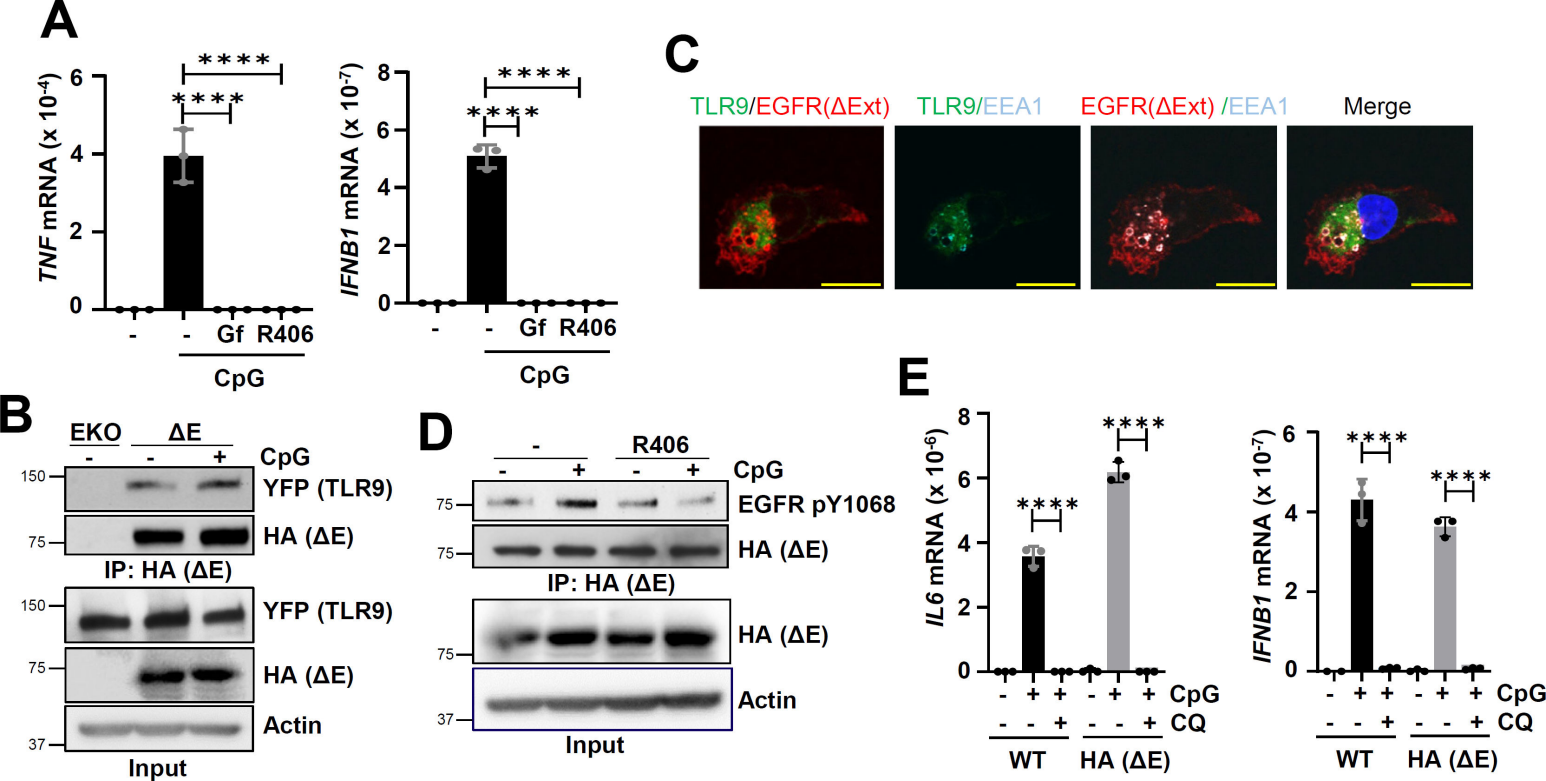
The ligand-binding extracellular domain of EGFR is not needed for TLR9-mediated gene induction. (**A**) hTLR9-HT1080 EGFR KO cells reconstituted with extracellular domain deleted EGFR (EGFRΔExt-HA, HA [ΔE]) were pre-treated with DMSO (-) or the EGFR inhibitor, gefitinib (Gf) (10 µM) or Syk inhibitor (R406) (10 µM) for 1 h and then treated with CpG ODN along with DMSO or Gf or R406 for 6 h and *TNF* and *IFNB1* mRNAs were measured by qRT-PCR. (**B**) HT1080 EGFR KO cells (EKO) and EKO cells reconstituted with EGFRΔExt-HA and hTRL9-YFP-Flag (ΔE) were treated with CpG ODN for 15 min, cell extracts were immunoprecipitated with anti-HA for EGFRΔExt (HA (ΔE)) and immunoblotted with anti-YFP for TLR9 and anti-HA for EGFRΔExt (HA [ΔE]). (**C**) HT1080 EGFR KO cells reconstituted with EGFRΔExt and hTRL9-YFP-flag were used for immunofluorescence. Red is EGFRΔExt, green is TLR9, cyan is for early endosomal marker, EEA1, and blue is for nucleus (scale bar = 10 µm). The first image is for TLR9/EGFR ΔExt interaction, the second is for TLR9/EEA1 interaction, the third is for EGFRΔExt/EEA1 interaction, and the fourth is for merge all with nucleus. (**D**) Cells, as in “**C**,” were pretreated with DMSO (-) or R406 for 1 h, followed by CpG ODN along with DMSO or R406 for 30 min, cell extracts were immunoprecipitated with anti-HA for EGFRΔExt (HA [ΔE]) and immunoblotted with anti-EGFR pY1068 and anti-HA for EGFRΔExt (HA [ΔE]). (**E**) WT and EGFRΔExt (HA [ΔE]) cells were pretreated with chloroquine (CQ) (100 µM) for 1 h before CpG ODN treatment along with CQ for 6 h; IL6 and IFNB1 mRNAs were measured by qRT-PCR. The data represent mean ± SD (**A and E**), ****P < 0.0001.

We enquired whether the ectodomain of EGFR, which binds EGF for its activation, was required for TLR9 signaling. To address this, we expressed a membrane-bound form of extracellular domain-deleted EGFR mutant (ΔE) in EGFR KO cells. The ΔE-expressing cells were able to induce TLR9-dependent genes, which were also suppressed by inhibitors of EGFR (Gf) or Syk (R406) (Fig. 4A). Since the cytoplasmic domain of EGFR was sufficient for TLR9-mediated gene induction, we evaluated whether ΔE binds TLR9. Indeed, the ΔE mutant, like the full-length EGFR, interacted with TLR9 either in the absence or the presence of CpG (Fig. 4B). We validated these results using confocal microscopy, which indicated that ΔE mutant co-localized with TLR9 (Fig. 4C). Furthermore, the TLR9-EGFR complex was endosomal, indicated by its colocalization with EEA1, an endosomal marker (Fig. 4C). Syk was required for CpG-mediated activation of full-length EGFR (Fig. 3E); ΔE mutant, which interacted with TLR9, was, as expected, inactive in the presence of Syk inhibitor, R406 (Fig. 4D). Endosomal acidification is a prerequisite for TLR9-dependent signaling (31); TLR9-mediated gene induction was inhibited by chloroquine (CQ), an inhibitor of endosomal acidification, in both Wt EGFR and ΔE-expressing cells (Fig. 4E). Collectively, our results indicated that the cytoplasmic domain of EGFR, which interacted with TLR9, was sufficient for TLR9-mediated gene induction. Furthermore, EGFR ectodomain, which is essential for EGF signaling, was dispensable for TLR9 functions.

### Mass spectrometric analyses revealed TLR9 is phosphorylated on Y^870^ and Y^980^ by Syk and EGFR, respectively

**Fig 5:**
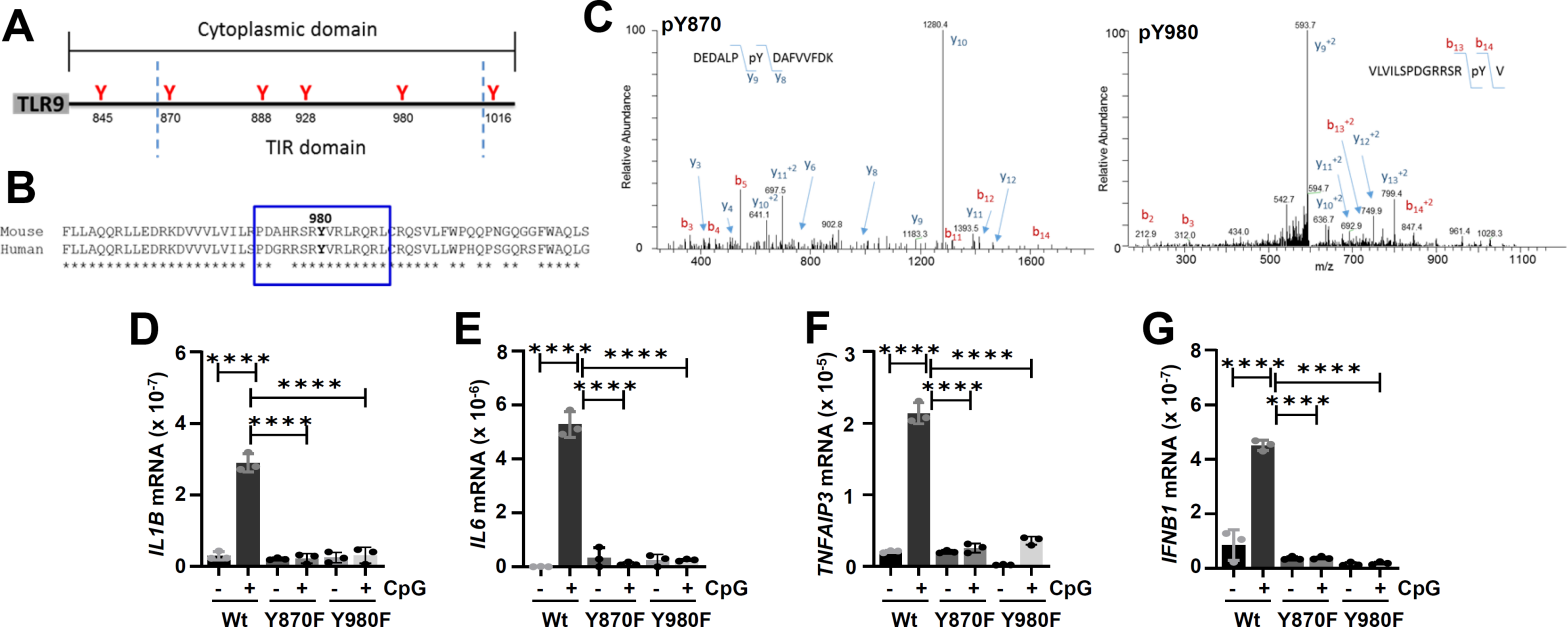
Two specific TLR9 tyrosine residues are phosphorylated after ligand stimulation. (**A**) Location of tyrosines in the TLR9 cytoplasmic domain and the TIR domain. (**B**) Predicted EGFR target tyrosine phosphorylation sites using GPS 6.0 in blue square. (**C**) 293XL-hTLR9-HA cells were treated with CpG ODN for 30 min and then immunoprecipitated with anti-HA for TLR9 to analyze for phosphorylation of the cytoplasmic TLR9 tyrosines using LC-MS/MS. Two phospho-peptides, DEDALPpYDAFVVFDK (pY870) and VLVILSPDGRRSRpYV (pY980) were identified. The MS/MS spectra for the phosphorylated peptides are shown. The doubly charged peptide (DEDALPpYDAFVVFDK) has an observed m/z of 932.9263 Da and is within 2.63 ppm of the expected mass. The masses of the y9 and y8 ions are consistent with phosphorylation at Y870. The triply charged peptide (VLVILSPDGRRSRpYV) has an observed m/z of 603.9943 Da and is within −2.81 ppm of the expected mass. The masses of the b14 and b13 ions are consistent with phosphorylation at Y980. (**D–G**) 293XL-hTLR9-YFP-flag (Wt), 293XL-hTLR9-YFP-flag Y870F (Y870F), and 293XL-hTLR9-YFP-flag Y980F (Y980F) cells were treated with CpG ODN for 6 h and induced *IL1B*, *IL6*, *TNFAIP3*, and *IFNB1* mRNAs were measured by qRT-PCR. The data represent mean ± SD (**D–G**), ****P < 0.0001.

Given the essential role of TLR9 Tyr phosphorylation in its activation, we determined the specific Tyr residues phosphorylated upon CpG treatment. TLR9 cytoplasmic domain contains six Tyr residues (Fig. 5A), and bioinformatic analyses revealed Y^980^ as a putative EGFR target site (Fig. 5B). To investigate whether Y^980^ is phosphorylated, we purified full-length TLR9 from CpG-treated cells and performed quantitative mass spectrometric analyses. Mass spectrometric analyses revealed the presence of two TLR9 cytoplasmic domain-specific phospho-peptides containing pY^870^ and pY^980^ (Fig. 5C). Mutation of either of these two Tyr residues (Y870F and Y980F) significantly inhibited TLR9-induced IL1B, IL6, TNFAIP3, and IFNB1 levels in CpG-treated cells (Fig. 5D through G). Mass spectrometry, followed byanalysesnal analyses, therefore, revealed that phosphorylation of Y^870^ and Y^980^ was essential for TLR9-mediated gene induction.

**Fig 6:**
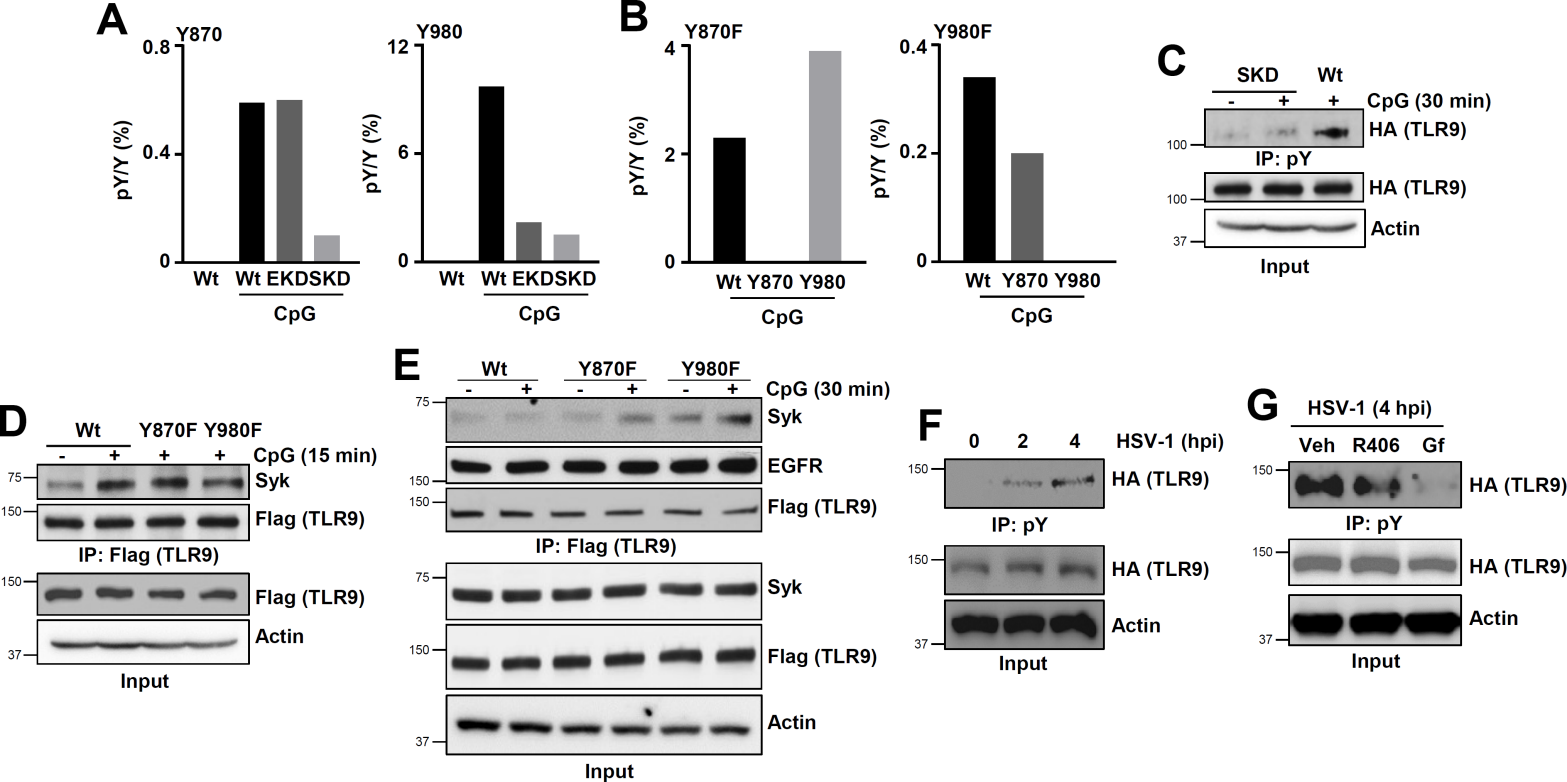
EGFR phosphorylates Y^980^ and Syk phosphorylates Y^870^ of TLR9 and the two phosphorylation events are independent of each other. (**A**) Wt, shSyk (SKD), and shEGFR (EKD) 293XL-hTLR9-HA cells were treated with CpG ODN for 30 min and then immunoprecipitated with anti-HA for TLR9 to analyze the phosphorylation of cytoplasmic TLR9 tyrosines using LC-MS/MS. The left panel shows the percentage peak area ratios of phospho-peptide and unmodified peptide (pY/Y %) for Y870 and the right panel for Y980. (**B**) 293XL-hTLR9-YFP-Flag (Wt), and TLR9 tyrosine mutants 293XL-hTLR9-YFP-flag Y870F (Y870F) and 293XL-hTLR9-YFP-flag Y980F (Y980F) cells were used. Treatment and analysis are as in “**A**.” The left panel shows pY/Y % for Y870 and the right panel for Y980 (**C**) 293XL-hTLR9-HA cells Wt and shSyk (SKD) were treated with CpG DNA for 30 min. Cell extracts were immunoprecipitated with anti-phospho tyrosine (pY) and immunoblotted with anti-HA for TLR9. (**D**) 293XL-TLR9-YFP-flag (Wt), 293XL-hTLR9-YFP-flag Y870F (Y870F), and 293XL-hTLR9-YFP-flag Y980F (Y980F) cells were treated with CpG ODN for 15 min. Cell extracts were immunoprecipitated with anti-flag for TLR9 and immunoblotted with anti-Syk and anti-flag for TLR9. (**E**) The indicated cells were treated with CpG ODN for 30 min. Cell extracts were immunoprecipitated with anti-flag for TLR9 and then immunoblotted with anti-Syk, anti-EGFR, and anti-flag for TLR9. (**F**) 293XL-hTLR9-HA cells were infected with HSV-1 (MOI:10) for the indicated times, then the cell lysates were immunoprecipitated with anti-pY antibody and immunoblotted with anti-HA antibody. (**G**) 293XL-hTLR9-HA cells were pre-treated with the inhibitors (R406 and Gf; 10 µM) for 2 h and then infected with HSV-1 as in F, and the cell lysates were immunoprecipitated with anti-pY antibody and immunoblotted with anti-HA antibody.

To determine whether EGFR and Syk phosphorylate these Tyr residues, we purified TLR9 from CpG-treated Wt, EKD, or SKD cells and performed quantitative mass spectrometry. Targeted analyses revealed that the relative abundance of pY^870^-containing phospho-peptide was reduced in SKD cells compared with Wt or EKD cells (Fig. 6A, left panel). In contrast, pY^980^-containing phospho-peptide abundance was reduced in both EKD and SKD cells compared with Wt cells (Fig. 6A, right panel). These results indicated that Y^870^ was likely phosphorylated by Syk, and Y^980^ was likely phosphorylated by EGFR. Moreover, since EGFR was not required for Syk recruitment to TLR9 (Fig. 3D), EKD cells showed pY^870^ (Fig. 6A, left panel). Whereas, since Syk activity was required for EGFR activation (Fig. 3E), SKD cells displayed reduced pY^980^ (Fig. 6A, right panel). To determine whether phosphorylation of these Tyr residues was interdependent, we used the TLR9 mutants (Y870F and Y980F) and performed quantitative mass spectrometry. As expected, the Y870F mutant completely abolished the abundance of pY^870^-containing phospho-peptide without affecting pY^980^ (Fig. 6B, the left panel). Similarly, pY^980^ signal was completely lost in the Y980F mutant but not in Y870F (Fig. 6B, right panel). Therefore, phosphorylation of the two Tyr was independent of each other. We confirmed the mass aspectrometric results using biochemical approach; SKD cells, lacking both pY^870^ and pY^980^ (Fig. 6A), exhibited reduced pTLR9, compared to Wt cells (Fig. 6C), demonstrating Syk as a new Tyr kinase phosphorylating TLR9. We further used TLR9 mutants to evaluate whether Syk recruitment to TLR9 required any of these two pY residues. Syk interacted with Wt as well as both Y870F and Y980F mutants of TLR9 with similar affinities (Fig. 6D), indicating phosphorylation of TLR9 was not a prerequisite for Syk recruitment. TLR9-EGFR interaction, as expected, was independent of TLR9 phosphorylation; both Y870F and Y980F mutants, like Wt TLR9, interacted similarly with EGFR (Fig. 6E). In the later phase, likely when TLR9 is completely phosphorylated, Syk was dissociated from TLR9 (Fig. 3A). However, partially phosphorylated TLR9, in Y870F or Y980F, could retain Syk in the later phase (Fig. 6E), further indicating phosphorylation of TLR9 led to Syk dissociation, presumably facilitating signalosome assembly. Next, we evaluated whether TLR9 gets tyrosine phosphorylated during virus infection and used HSV-1 infection model. HSV-1 infection caused time-dependent phosphorylation of TLR9 in Wt cells (Fig. 6F). Moreover, pre-treatment with Syk or EGFR inhibitors led to inhibition of TLR9 tyrosine phosphorylation (Fig. 6G). Together, our results demonstrated that the TLR9 cytoplasmic domain was phosphorylated sequentially by Syk and EGFR on Y^870^ and Y^980^, respectively, to achieve a fully functional TLR9.

### CpG DNA activates Syk by scavenger receptor A and Lyn, independent of TLR9

**Fig 7:**
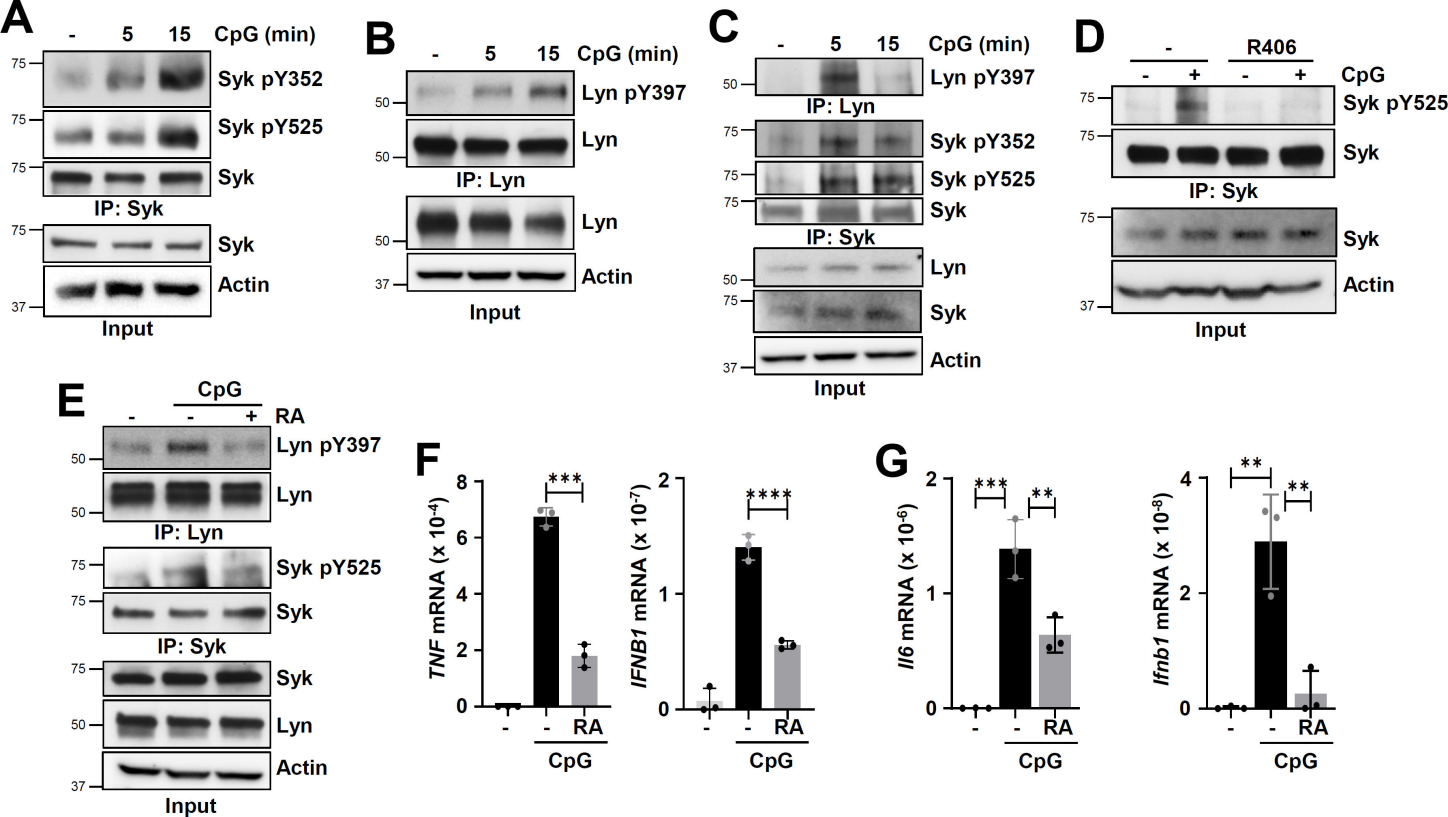
Scavenger receptor A is required for CpG-activated Syk and TLR9-induced genes. (**A and B**) 293XL cells were treated with CpG DNA (10 µg/mL) for different lengths of time, and the cell extracts were subjected to immunoprecipitation (IP) followed by immunoblot as indicated. (**C**) 293XL-hTLR9-HA cells were treated with CpG ODN (10 µg/mL) for the indicated time and then immunoprecipitated (IP) with anti-Lyn or anti-Syk and immunoblotted with the indicated antibodies. (**D**) 293XL-hTLR9-HA cells were pre-treated for 15 min with the Syk inhibitor, R406, or the vehicle (DMSO) and then treated with CpG ODN along with R406 or DMSO for 15 min. The cell extracts were immunoprecipitated with anti-Syk and immunoblotted with anti-Syk pY525 and anti-Syk. (**E**) 293XL cells were pretreated with DMSO (-) or scavenger receptor-A (SR-A) inhibitor, rhein (RA) (100 µM) for 2 h and then treated for 15 min with CpG ODN, along with DMSO. The cell extracts were immunoprecipitated with anti-Lyn or anti-Syk and immunoblotted with anti-Lyn pY397, anti-Syk pY352, anti-Lyn, and anti-Syk. (**F**) 293XL-hTLR9-HA cells were pretreated with DMSO or scavenger receptor-A (SR-A) inhibitor, rhein (RA) (100 µM) for 2 h and then treated with CpG ODN along with DMSO or RA for 6 h, and induced *TNF* and *IFNB1* mRNAs were measured by qRT-PCR. (**G**) RAW264.7 cells were treated and analyzed as in “E” for induced *Il6* and *Ifnb1* mRNAs by qRT-PCR. The data represent mean ± SD (**F and G**), **P < 0.01, ***P < 0.001, ****P < 0.0001.

Given Syk’s essential role in phosphorylating TLR9, we investigated the mechanism of its activation in CpG-treated cells. CpG treatment caused rapid activation of Syk, analyzed by its phosphorylation on Y^352^ and Y^525^, in HEK cells (Fig. 7A). Kinase activation of Syk requires Lyn-mediated pY^352^, followed by autophosphorylation on Y^525^, which was also observed in CpG-treated cells (Fig. 7A). Lyn, the Syk-activating Tyr kinase, was also activated rapidly in CpG-treated HEK cells (Fig. 7B). To investigate the role of TLR9 in Syk and Lyn activation, we used HEK-TLR9 cells, in which CpG treatment also caused rapid activation of Lyn and Syk (Fig. 7C). Lyn activation in these cells, however, was transient; it was dephosphorylated 15 min post-CpG-treatment (Fig. 7C). R406, a Syk kinase inhibitor, blocked pY^525^, indicating CpG treatment caused autophosphorylation, which is required for activation of Syk (Fig. 7D). Since Syk was activated by CpG treatment in both HEK and HEK-TLR9 cells, its activation was TLR9-independent. To determine the receptor activating Lyn and Syk in the absence of TLR9, we examined scavenger receptor A (SR-A), a pattern recognition receptor on the plasma membrane, which binds CpG and activates SFKs in myeloid cells (32, 33). We used Rhein (RA), a chemical inhibitor of SR-A, to examine TLR9-independent CpG-induced pLyn and pSyk. CpG-induced pLyn and pSyk were inhibited by RA in HEK cells, indicating SR-A was involved in activation of Lyn and Syk (Fig. 7E). Since SR-A was required for CpG-activated Lyn and Syk, we evaluated its role in TLR9-induced genes. RA treatment caused significant inhibition of TLR9-induced TNF and IFNB1 in HEK-TLR9 cells (Fig. 7F). The role of SR-A was further evaluated in mouse myeloid cells; RA treatment also inhibited TLR9-induced Il6 and Ifnb1 in RAW264.7 macrophages (Fig. 7G). Together, these results revealed a new role of SR-A in CpG-mediated activation of Syk, which is essential for phosphorylating TLR9 and downstream signaling.

### Syk, Lyn, EGFR, and SR-A are required for TLR9-induced gene expression in primary and immortalized myeloid cells

**Fig 8:**
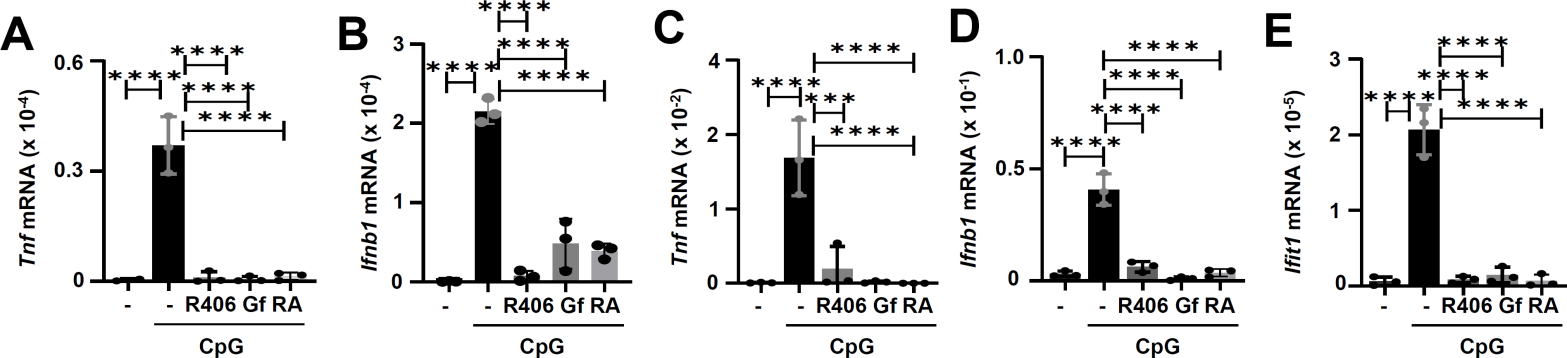
Inhibitors of Syk, EGFR, and scavenger receptor A suppress TLR9-induced gene expression in primary cells. (**A and B**) Primary BMDMs were either untreated or treated with Syk inhibitor (R406), EGFR inhibitor (Gf), scavenger receptor-A (SR-A) inhibitor, rhein (RA), or the vehicle (DMSO) and then treated with CpG ODN (10 µg/mL) for 6 h. The induction of *Tnf* and *Ifnb1* was analyzed by qRT-PCR. (**C–E**) Primary splenocytes were either untreated or treated with CpG DNA (10 µg/mL) in the absence or presence of R406, Gf, or RA. The induction of *Tnf*, *Ifnb1*, and *Ifit1* was analyzed by qRT-PCR. The data represent mean ± SD, ***P < 0.001, ****P < 0.0001.

**Fig 9:**
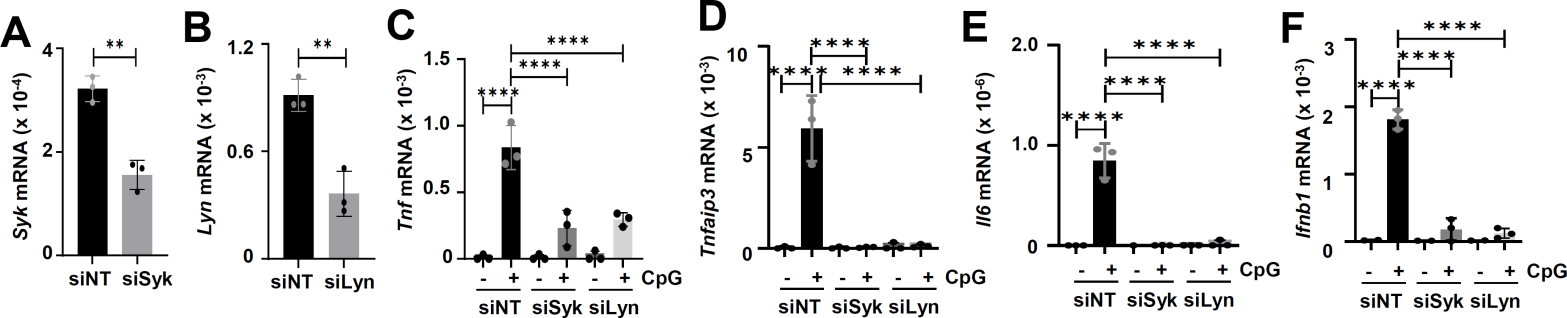
Knockdown of Syk and Lyn suppresses TLR9-induced gene expression in macrophages. (**A and B**) RAW264.7 mouse macrophages were transfected with siRNAs against Syk (siSyk), Lyn (siLyn), or a non-targeting (siNT) control. The levels of *Syk* and *Lyn* mRNAs were analyzed by qRT-PCR. (**C–F**) RAW264.7 mouse macrophages were transfected with Syk and Lyn siRNAs and treated with CpG ODN, as indicated. The mRNA levels of *tnf*, *Tnfaip3*, *Il6,* and *Ifnb1* were analyzed by qRT-PCR. The data represent mean ± SD, **P < 0.01, ****P < 0.0001.

We finally evaluated the roles of the newly identified components of TLR9 signaling in mouse myeloid cells. In primary BMDMs, the inhibitors of Syk (R406), EGFR (Gf), and SR-A (RA) strongly suppressed the TLR9-induced Tnf and Ifnb1 genes (Fig. 8A and B). Similar to primary BMDMs, in primary splenocytes, these inhibitors strongly suppressed the TLR9-induced Tnf, Ifnb1, and Ifit1 genes (Fig. 8C through E). We validated the results using pharmacological agents by genetic approach and knocked down Syk and Lyn in RAW264.7 mouse macrophage cell line (Fig. 9A and B). As expected, in these cells, the knockdown of Syk and Lyn also inhibited the TLR9-induced Tnf, Tnfaip3, IL6, and Ifnb1 gene expression (Fig. 9C through F). Together, our results from primary and immortalized myeloid cells demonstrate the functional requirements of Syk, Lyn, EGFR, and SR-A in TLR9 signaling.

## DISCUSSION

**Fig 10:**
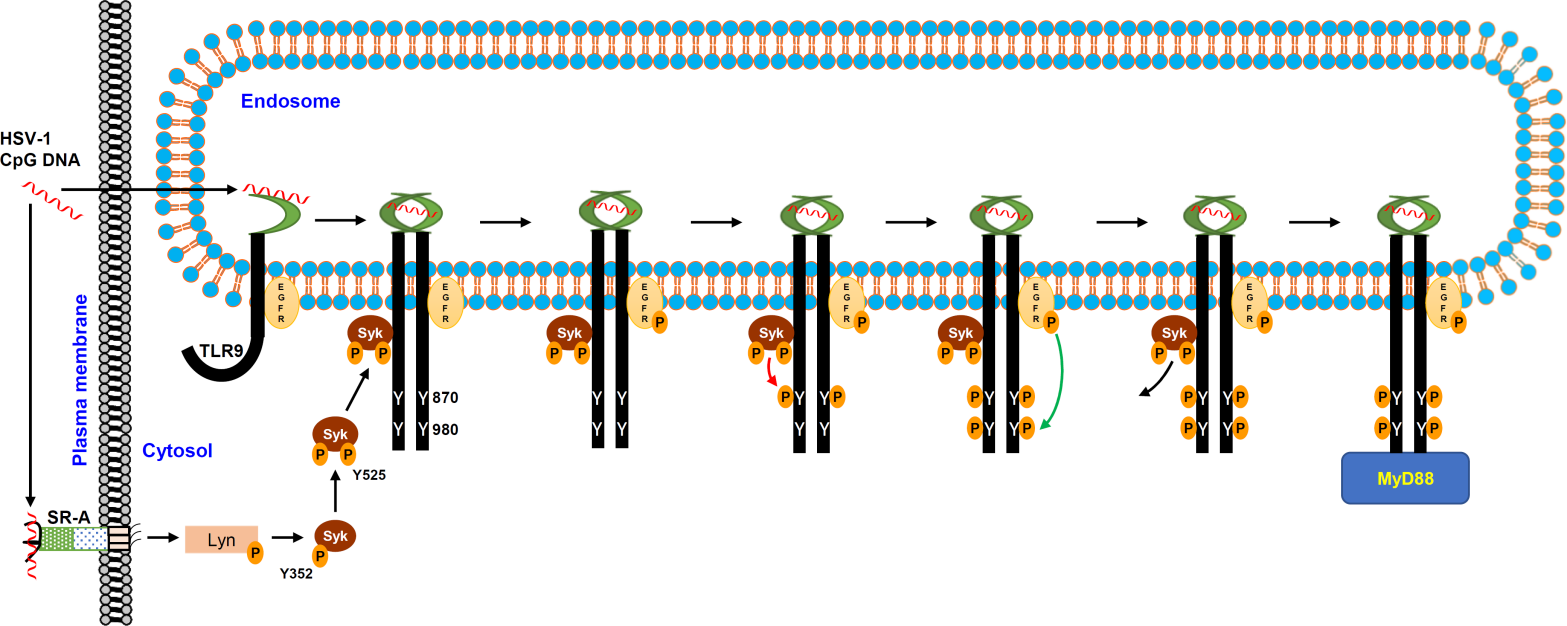
TLR9 is phosphorylated by EGFR and Syk for its activation. CpG DNA stimulation causes SR-A-mediated activation of Lyn, which phosphorylates Syk. Phosphorylated Syk (p-Syk) further autophosphorylates itself to form the activated Syk that gets recruited to endosomal TLR9, which binds EGFR constitutively. Syk recruitment to TLR9 activates EGFR by autophosphorylation; activated Syk and EGFR sequentially phosphorylate TLR9 on Y870 and Y980, respectively. Doubly phosphorylated TLR9 acts as a fully activated receptor for intracellular signaling via MyD88.

In this study, we reveal the molecular details of the early stages of ligand-mediated TLR9 activation (Fig. 10). Previously, we showed that TLR9 binds to an endosomal pool of EGFR, a tyrosine kinase, constitutively, enabling it to phosphorylate the cytoplasmic domain of TLR9 upon CpG stimulation (30). In the current study, we demonstrated that TLR9-bound EGFR required phosphorylation, an activation signal mediated by another tyrosine kinase, Syk. Syk was recruited to TLR9 upon CpG stimulation, and Syk kinase activity was essential for activating TLR9-bound EGFR. These results led us to postulate that TLR9 full activation required phosphorylation by EGFR and Syk. Quantitative mass spectrometric analyzes revealed Syk and EGFR sequentially phosphorylated Y^870^ and Y^980^ of TLR9 cytoplasmic domain, respectively. Mutation of either of these two Tyr residues led to incomplete activation of TLR9, resulting in the inhibition of TLR9-mediated gene induction. Unlike EGFR, Syk recruitment to TLR9 required its phosphorylation, mediated partially by Lyn, a cytosolic tyrosine kinase. Lyn was activated, surprisingly, independent of TLR9, by a membrane-bound scavenger receptor, SR-A, upon CpG binding. Our results, obtained from human cell model, primary and immortalized mouse myeloid cells, led to a two-step activation model for TLR9 mediated by two tyrosine kinases, Syk and EGFR.

TLRs remain in closed conformation to maintain inactive states to avoid undesired activities. Activation of TLRs requires their posttranslational modifications, often mediated by Tyr phosphorylation (25). Upon binding its ligand, dsRNA, generated during viral replication or tissue damage, the cytoplasmic domain of TLR3 gets phosphorylated on two Tyr residues (29). Two Tyr kinases, EGFR and Src, phosphorylate Y^858^ and Y^759^ of TLR3 cytoplasmic domain, respectively. Phosphorylation of these residues results in the recruitment of downstream adaptor protein, TRIF, which, via a series of signaling proteins, activates IRF3 and NF-κB, to induce antiviral and inflammatory genes. In contrast, TLR4 signaling requires EGFR to activate IRF3 but not NF-κB (34). TLR9, on the other hand, required EGFR and Syk for phosphorylating Y^980^ and Y^870^, respectively, for full activation. These studies indicate the EGFR requirement for endosomal TLRs. A structural role of Y^870^ for TLR9 has also been shown to be independent of tyrosine phosphorylation (35). Mutation of Y^870^ causes immature processing of TLR9, leading to impaired downstream signaling. Recent studies further suggest that EGFR is required for cytoplasmic DNA-activated cGAS/STING signaling pathway (36, 37). Ligand-activated STING interacts with EGFR, which then phosphorylates Y^245^ of STING. STING phosphorylation is required for its intracellular trafficking, a step critical for its downstream signaling. EGFR, however, is not needed for cytoplasmic RNA sensors, the RIG-I-like receptors (RLRs), indicating its specificity for endosomal TLRs and STING, which are transmembrane proteins. EGFR, therefore, is a potential therapeutic target for diseases regulated by these PRRs.

An unexpected finding from our studies was CpG-mediated activation of Syk by SR-A and independent of TLR9. Scavenger receptors function as co-activators of multiple TLRs to help facilitate ligand binding. We report a novel crosstalk between TLR9 and SR-A mediated by Lyn and Syk. SR-A, upon recognition of CpG ODN, activated Lyn, which further led to the activation of Syk, a kinase for TLR9. The role of SR-A in the context of sensing viral infection remains unclear. The cell surface SR-A molecules may recognize DAMPs released from infected cells and can synergize with partially activated TLR9, which senses pathogen-derived DNA. SR-A1 can sense HCMV viral proteins, further synergizing with endosomal TLR3 and TLR9 signaling (38). A role of SR-A has also been shown in the context of viral-bacterial co-infection, in which TLR9 acts as a negative regulator (39). Furthermore, it is possible that SR and TLR proteins can interact to facilitate signaling outcomes (40). The requirement of Lyn in the context of TLR9 signaling was primarily to activate Syk, the executioner kinase. It would be interesting to see if B cells, major responders of TLR9 agonists, might utilize BCR-activated Syk, independent of SR-A. Previously, functional requirements of Lyn and Hck, two SFKs, have been implicated in TLR9-induced gene expression in macrophages. This study also reported a two-step activation model of TLR9 signaling, in which Syk was shown to interact with TLR9 in CpG-activated human macrophage (41). Our current study stems from these critical results and adds new molecular details to the TLR9 activation mechanism. Antigen-activated BCR recruits Syk, which may function as a TLR9 kinase for IRF and NF-κB activation (42). CpG-induced Syk activation has been shown before in B cells; however, this was dependent on TLR9. Syk, therefore, may have cell type-specific activation mechanisms in TLR9 signaling. A role of Syk in TLR9 signaling has previously been described in fungal infection (43). Dectin-1, a receptor for fungal cell wall carbohydrates, can activate Syk, which facilitates TLR9 processing and trafficking to the phagosome. In this context, whether Dectin-1-activated Syk also phosphorylates TLR9 remains to be investigated. An anti-apoptotic function of TLR9 was observed in *L. donovani*-infected macrophages (44). *L. donovani* DNA activates TLR9, which, upon recruitment of Syk, can delay the macrophage apoptosis. Syk, therefore, can regulate various functions of TLR9 context dependently.

In addition to Syk, TLR9 itself or components of the TLR9 signaling complex can be phosphorylated by other Tyr kinases cell-specifically. Bruton’s Tyrosine Kinase (BTK) plays a key role in TLR9 functions either by targeting TLR9 directly or proteins involved in TLR9-mediated intracellular signaling. A plasmacytoid dendritic cell (pDC) specific role of BTK has been characterized, specifically for TLR9 but not TLR7 functions (45). BTK inhibitors specifically block TLR9-mediated interferon and inflammatory responses in pDCs. BTK has been shown to mediate a synergistic function in B cells by allowing TLR9 and BCR colocalization in cellular autophagosome-like compartments (46). BTK is required for synergistic cytokine response by TLR9 and BCR in human and murine B cells, with implications in B cell activation and autoimmune diseases. DOCK8, an adaptor of the TLR9 signaling pathway in B cells, gets Tyr phosphorylated by Pyk2, and the phosphorylation is essential for its interaction with TLR9. DOCK8 phosphorylation is required for TLR9-activated Src-Syk-STAT3 signaling cascade, essential for B-cell proliferation and immunoglobulin production.

In summary, our studies provide new insights into the complete activation process of TLR9 that is essential for downstream signaling. We identified Syk, Lyn, and EGFR as key kinases involved in this activation, which may serve as potential therapeutic targets for modulating TLR9-mediated pathogenic responses. Whether these kinases are functionally altered in diseases regulated by TLR9 remains to be determined. Additionally, the newly uncovered role of SR-A in TLR9 activation opens avenues for exploring crosstalk between these signaling pathways.

## MATERIALS AND METHODS

### Reagents and antibodies

Syk inhibitor (inh-r406), R406 was from Invivogen, Gefitinib (S1025) and Scavenger receptor A inhibitor (Rhein, s2400) were obtained from Selleckchem, and CpG oligonucleotide (CpG-B) was from Integrated DNA Technologies (IDT). TLR9 antibody (mAb-mTLR9) obtained from Invivogen. Phospho-tyrosine antibody (05-321) was obtained from Millipore, antibodies against EGFR (2646), phospho-EGFR Y1068 (2234), MyD88 (4283), Syk (13198, 80460), phospho-Syk Y352 (2701), phospho-Syk Y525 (2711), Lyn (2796, 4576), and actin (A5441) were from Cell Signaling Technology, anti-phospho Lyn Y397 (orb315590) was from Biorbyt, HA antibody (ab18181, ab182009) was from Abcam, Chloroquine (C6628), anti-flag M2 affinity gel (A2220), and anti-flag antibody (F7425, F1804) were from Sigma Aldrich, anti-YFP (SC-32897) and anti-Scavenger receptor-A (SR-A, SC-166184) from Santa Cruz Biotechnology.

### Cell lines

Primary bone marrow-derived macrophages (BMDMs) or splenocytes were isolated from Wt C57BL/6 mice (JAX) and differentiated as described before (30, 47, 48). All animal procedures were performed using approved IACUC protocol at the University of Kentucky. RAW264.7 cells from ATCC were maintained in DMEM containing 10% FBS and penicillin-streptomycin. 293XL and 293XL-human TLR9-HA cells from Invivogen were maintained in DMEM containing 10% FBS, penicillin-streptomycin, normocin, and blasticidin as per manufacturer’s instructions. 293XL and HT1080 cells with human TLR9 (with YFP and Flag double epitope tags), TLR9, Y870F, and Y980F were generated using lentiviral transduction. EGFR-knockdown, Syk-knockdown, and Lyn-knockdown in 293XL-human TLR9-HA cells were generated by lentiviral transduction of human EGFR-specific shRNA (Sigma, #TRCN0000121202), Syk-specific shRNA (Sigma, #TRCN0000197242), and Lyn-specific shRNA (Sigma, # TRCN0000218210), respectively, followed by selection under puromycin and expressions were confirmed using western blot (WB). A non-targeting shRNA (#SHC002) was used as a control. SiRNAs against Syk and Lyn were obtained from Horizon Discovery and transfected using the manufacturer’s instructions. The cells were treated with CpG after 48 h of transfection. The CRISPR-induced genomic deletion was performed by overnight lentivirus transduction of sub-confluent HT1080 cells LentiCRISPRv2 expressing Cas-9 endonuclease and Human EGFR sgRNA sequences TGAGCTTGTTACTCGTGCCT and GAGTAACAAGCTCACGCAGT were used. The knockout cells were then selected under puromycin. Single-cell clones were screened using genomic DNA sequencing and WB. The extracellular domain deleted EGFR (Δext) expression vector was kindly provided by Xiaoxia Li.

### Quantitative real-time PCR

Total cellular RNA was extracted from cells using Roche RNA isolation kit (11828665001, Roche), and used for cDNA preparation using Impromp-II Reverse Transcription Kit (A3803, Promega). The cDNA (0.5 ng) was applied to a 384-well plate for real-time PCR using SYBR Green PCR mix (Applied Biosystem’s Power) in Roche Light Cycler 480 II. The expression levels of the induced mRNAs were normalized to 18S rRNA or RPL32 mRNA. GraphPad Prism was used for plotting.

### Immunoprecipitation and Western blot

Cells were lysed on ice using 50 mM Tris–HCl pH 7.4 containing 150 mM NaCl, 5 mM EDTA, 1 mM DTT, 1 mM PMSF, and cocktail (Roche) for Western blot and 20 mM HEPES pH 7.5 containing 150 mM NaCl, 10 mM NaF, 1.5 mM MgCl2, 10 mM β‐glycerophosphate, 2 mM EGTA, 1 mM sodium orthovanadate, 0.5% (v/v) Triton X‐100, and protease inhibitors (Roche Applied Science, Indianapolis, IN, USA). The lysates were pre-cleared with A/G agarose beads. The pre-cleared lysates were then treated with antibodies to targeted proteins for two hours at 4°C. These were then incubated with A/G agarose beads (426535, Santa Cruz Biotechnology) overnight at 4°C. The beads obtained after centrifugation were washed thrice with lysis buffer and boiled with SDS-PAGE buffer to elute the proteins. The eluted samples were then analyzed by SDS-PAGE followed by Western blot. The samples were run through the SDS-PAGE gels and then transferred to the polyvinylidene difluoride (PVDF) membrane (Bio-Rad). The membranes were then incubated for 1 h in 5% skim milk in TBST buffer (150  mM NaCl; Tris, pH 7.4; and 0.1% Tween 20) at room temperature. The membranes were then incubated with the primary antibody in a cold room overnight, followed by the secondary HRP antibody. The membranes were then visualized by Super-Signal West Pico chemiluminescent substrate (Pierce Chemical) and shown representatives of three independent experiments.

### Mass spectrometry

After treatment or non-treatment, the biological triplicate cells were pooled and lysed, pulled down with anti-HA or anti-flag, and then separated using SDS-PAGE. Protein digestions were done using the TLR9 bands from the gels, after washing and de‐staining in 50% ethanol containing 5% acetic acid, the gel pieces were dehydrated in acetonitrile, dried in a Speed‐vac, and digested by adding 5 µL 10 ng/µL of trypsin (to identify Y870, Y887, and Y928), alpha-lytic (to identify Y845 and Y928), and trypsin-AspN (Y980 and Y1021), in 50 mM ammonium bicarbonate. After overnight incubation, the peptides were extracted into two portions of 30 µL each 50% acetonitrile and 5% formic acid. The combined extracts were then evaporated to ~10 µL in a Speed‐vac and re‐suspended in 1% acetic acid to make up to 30 µL for analysis.

Finnigan LTQ‐Orbitrap Elite hybrid mass spectrometer was used for LC-MS study for hTLR9-HA cells (Fig. 5 and 6A) with Dionex 15 cm × 75 µm id Acclaim PepMap C18, 2 µm, 100 Å reversed‐phase capillary chromatography column. The micro‐electrospray ion source was operated at 2.5 kV. Then, 5 µL of the extract was injected into the LC-MS system, and the peptides eluted from the column, using an acetonitrile/0.1% formic acid gradient at a flow rate of 0.25 µL/min, were introduced into the source of the mass spectrometer online. The digest was analyzed in both a survey manner and a targeted manner. The survey experiments were performed using the data‐dependent multitask capability of the instrument, acquiring full scan mass spectra to determine peptide molecular weights and product ion spectra to determine amino acid sequences in successive instrument scans. The LC‐MS/MS data were then searched using Mascot and Sequest programs against the full human reference sequence database, specifically against the sequence of TLR9. The parameters used in this search include a peptide mass accuracy of 10 ppm, fragment ion mass accuracy of 0.6 Da, carbamidomethylated cysteines as a constant modification, and oxidized methionine and phosphorylation at S, T, and Y as a dynamic modification. The results were then filtered based on Mascot ion scores and Sequest Xcorr scores. All positively identified phosphopeptides were manually validated. The targeted experiments involve the analysis of specific TLR9 peptides, including the phosphorylated and unmodified forms of the Y845, Y870, Y888, Y928, Y980, and Y1021 peptides. The chromatograms for these peptides were then plotted based on known fragmentation patterns. The peak areas of these chromatograms were used to determine the extent of phosphorylation (49).

hTLR9-YFP samples (Fig. 6B) were analyzed by LC-MS using a Fusion Lumos Tribrid MS (Thermo Scientific) equipped with a Dionex Ultimate 3000 nano UHPLC system, and a Dionex (25 cm × 75 µm id) Acclaim Pepmap C18, 2 µm, 100 Å reversed-phase capillary chromatography column. Peptide digests (5 µL) were injected into the reverse phase column and eluted at a flow rate of 0.3 µL/min using mobile phase A (0.1% formic acid in H2O) and B (0.1% formic acid in acetonitrile). The gradient was held at 2% B for 5 min, %B was increased linearly to 35% in 80 min, increased linearly to 90% B in 10 min, and maintained at 90% B for 5 min. The mass spectrometer was operated in a data-dependent manner, which involved full scan MS1 (375-1700 Da) acquisition in the Orbitrap MS at a resolution of 120,000. This was followed by CID (1.6 Da isolation window) at 35% CE and ion trap detection. MS/MS spectra were acquired for 3 s. The second method was used for glycopeptide identification and involved full scan MS1 7 (350–1,700 Da) acquisition in the Orbitrap MS at a resolution of 120,000. Dynamic exclusion was enabled where ions within 10 ppm were excluded for 60 s.

### Immunofluorescence and confocal microscopy

EGFR KO HT1080-TLR9 (with YFP and Flag double epitope tags) cells, reconstituted with extracellular domain deleted EGFR, were grown on glass coverslips, and the cells were fixed for 20 min with 4% paraformaldehyde and permeabilized with 0.2% Triton X-100. Fixed cells were blocked with 5% normal goat serum for 1 h and labeled overnight with anti-EEA1 (610457, BD Transduction Labs) to stain early endosomes and anti-EGFR (2646, Cell Signaling). Goat anti-mouse Alexa Fluor 647 (A32728, Invitrogen) and anti-rabbit Alexa Fluor 594 (A32740, Invitrogen) (for 1 h) were used, respectively, as secondary antibodies. These were then mounted using VECTASHIELD-DAPI. The images were acquired by confocal laser scanning microscopy (Leica TCS SP8) using oil immersion ×63 objective and were processed with Leica LCS software.

### *In situ* proximity ligation assay (PLA)

Previously described procedures were used for PLA as per the manufacturer’s instructions (30). Briefly, cells were grown on glass coverslips and did the treatment. They were then fixed using 4% paraformaldehyde followed by permeabilization using 0.5% Triton X, blocking with 2.5% goat serum, and incubated overnight with TLR9 and MyD88 primary antibodies. The coverslips were then incubated for 1 h at 37°C with Duolink *In situ* PLA Probe Anti-Mouse MINUS (DUO92004, Sigma Aldrich) and Duolink *In situ* PLA Probe Anti-Rabbit PLUS (DUO92002, Sigma-Aldrich) in 2.5% goat serum. These were then incubated with DNA ligase and DNA polymerase and then mounted in VectaShield/DAPI. Images were acquired using a Zeiss confocal microscope. The PLA dots were counted using Fiji software.

### Phospho-tyrosine assay for TLR9 by HSV-1 infection

HEK-TLR9 cells were infected with HSV-1 KOS strain (MOI:10) for 2 and 4 h using previously described procedures, and then the cells were lysed using the above-mentioned lysis buffer. The cell lysates were then used for immunoprecipitation with anti-phosphotyrosine magnetic beads (Sigma#16-282, clone 4G10), and the immunoprecipitates were analyzed by immunoblot with anti-HA antibody.

### Statistical analysis

GraphPad Prism 9 software was used for all statistical analyses. Biological replicates were used to present the qRT-PCR results; *P*-values were calculated using one-way ANOVA for more than two groups and unpaired Student’s *t*-tests for two groups.

## Data Availability

All data presented in this paper are contained within the manuscript.

## References

[1] Fensterl V, Chattopadhyay S, Sen GC. 2015. No love lost between viruses and interferons. Annu Rev Virol 2:549–572. doi:10.1146/annurev-virology-100114-055249 26958928 PMC9549753

[2] Lee MS, Kim YJ. 2007. Signaling pathways downstream of pattern-recognition receptors and their cross talk. Annu Rev Biochem 76:447–480. doi:10.1146/annurev.biochem.76.060605.122847 17328678

[3] Li D, Wu M. 2021. Pattern recognition receptors in health and diseases. Signal Transduct Target Ther 6:291. doi:10.1038/s41392-021-00687-0 34344870 PMC8333067

[4] Takeuchi O, Akira S. 2010. Pattern recognition receptors and inflammation. Cell 140:805–820. doi:10.1016/j.cell.2010.01.022 20303872

[5] Yu L, Liu P. 2021. Cytosolic DNA sensing by cGAS: regulation, function, and human diseases. Signal Transduct Target Ther 6:170. doi:10.1038/s41392-021-00554-y 33927185 PMC8085147

[6] Glanz A, Chakravarty S, Varghese M, Kottapalli A, Fan S, Chakravarti R, Chattopadhyay S. 2021. Transcriptional and non-transcriptional activation, posttranslational modifications, and antiviral functions of interferon regulatory factor 3 and viral antagonism by the SARS-coronavirus. Viruses 13:575. doi:10.3390/v13040575 33805458 PMC8066409

[7] Villalón-Letelier F, Brooks AG, Saunders PM, Londrigan SL, Reading PC. 2017. Host cell restriction factors that limit influenza A infection. Viruses 9:376. doi:10.3390/v9120376 29215570 PMC5744151

[8] Martin-Sancho L, Lewinski MK, Pache L, Stoneham CA, Yin X, Becker ME, Pratt D, Churas C, Rosenthal SB, Liu S, et al.. 2021. Functional landscape of SARS-CoV-2 cellular restriction. Mol Cell 81:2656–2668. doi:10.1016/j.molcel.2021.04.008 33930332 PMC8043580

[9] Zhou Y, He C, Wang L, Ge B. 2017. Post-translational regulation of antiviral innate signaling. Eur J Immunol 47:1414–1426. doi:10.1002/eji.201746959 28744851 PMC7163624

[10] Popli S, Chakravarty S, Fan S, Glanz A, Aras S, Nagy LE, Sen GC, Chakravarti R, Chattopadhyay S. 2022. IRF3 inhibits nuclear translocation of NF-κB to prevent viral inflammation. Proc Natl Acad Sci USA 119:e2121385119. doi:10.1073/pnas.2121385119 36067309 PMC9478676

[11] Fan S, Popli S, Chakravarty S, Chakravarti R, Chattopadhyay S. 2024. Non-transcriptional IRF7 interacts with NF-κB to inhibit viral inflammation. J Biol Chem 300:107200. doi:10.1016/j.jbc.2024.107200 38508315 PMC11040127

[12] Osterlund PI, Pietilä TE, Veckman V, Kotenko SV, Julkunen I. 2007. IFN regulatory factor family members differentially regulate the expression of type III IFN (IFN-lambda) genes. J Immunol 179:3434–3442. doi:10.4049/jimmunol.179.6.3434 17785777

[13] Dalskov L, Gad HH, Hartmann R. 2023. Viral recognition and the antiviral interferon response. EMBO J 42:e112907. doi:10.15252/embj.2022112907 37367474 PMC10350828

[14] Atarashi N, Morishita M, Matsuda S. 2024. Activation of innate immune receptor TLR9 by mitochondrial DNA plays essential roles in the chemical long-term depression of hippocampal neurons. J Biol Chem 300:105744. doi:10.1016/j.jbc.2024.105744 38354781 PMC10943477

[15] Tripathi A, Bartosh A, Whitehead C, Pillai A. 2023. Activation of cell-free mtDNA-TLR9 signaling mediates chronic stress-induced social behavior deficits. Mol Psychiatry 28:3806–3815. doi:10.1038/s41380-023-02189-7 37528226 PMC10730412

[16] Uematsu S, Sato S, Yamamoto M, Hirotani T, Kato H, Takeshita F, Matsuda M, Coban C, Ishii KJ, Kawai T, Takeuchi O, Akira S. 2005. Interleukin-1 receptor-associated kinase-1 plays an essential role for toll-like receptor (TLR)7- and TLR9-mediated interferon-{alpha} induction. J Exp Med 201:915–923. doi:10.1084/jem.20042372 15767370 PMC2213113

[17] Wong W. 2011. UnPINning IRAK1. Sci Signal 4:ec203–ec203. doi:10.1126/scisignal.4183ec203

[18] Majer O, Liu B, Woo BJ, Kreuk LSM, Van Dis E, Barton GM. 2019. Release from UNC93B1 reinforces the compartmentalized activation of select TLRs. Nature 575:371–374. doi:10.1038/s41586-019-1611-7 31546247 PMC6856438

[19] Pelka K, Bertheloot D, Reimer E, Phulphagar K, Schmidt SV, Christ A, Stahl R, Watson N, Miyake K, Hacohen N, Haas A, Brinkmann MM, Marshak-Rothstein A, Meissner F, Latz E. 2018. The chaperone UNC93B1 regulates toll-like receptor stability independently of endosomal TLR transport. Immunity 48:911–922. doi:10.1016/j.immuni.2018.04.011 29768176 PMC6482051

[20] Song HS, Park S, Huh JW, Lee YR, Jung DJ, Yang C, Kim SH, Kim HM, Kim YM. 2022. N-glycosylation of UNC93B1 at a specific asparagine residue is required for TLR9 signaling. Front Immunol 13:875083. doi:10.3389/fimmu.2022.875083 35874766 PMC9301129

[21] Hao L, Zhong W, Sun X, Zhou Z. 2021. TLR9 signaling protects alcohol-induced hepatic oxidative stress but worsens liver inflammation in mice. Front Pharmacol 12:709002. doi:10.3389/fphar.2021.709002 34262465 PMC8273378

[22] Garcia-Martinez I, Santoro N, Chen Y, Hoque R, Ouyang X, Caprio S, Shlomchik MJ, Coffman RL, Candia A, Mehal WZ. 2016. Hepatocyte mitochondrial DNA drives nonalcoholic steatohepatitis by activation of TLR9. J Clin Invest 126:859–864. doi:10.1172/JCI83885 26808498 PMC4767345

[23] Fillatreau S, Manfroi B, Dörner T. 2021. Toll-like receptor signalling in B cells during systemic lupus erythematosus. Nat Rev Rheumatol 17:98–108. doi:10.1038/s41584-020-00544-4 33339987 PMC7747191

[24] Diskin C, Ryan TAJ, O’Neill LAJ. 2021. Modification of proteins by metabolites in immunity. Immunity 54:19–31. doi:10.1016/j.immuni.2020.09.014 33220233

[25] Chattopadhyay S, Sen GC. 2014. Tyrosine phosphorylation in toll-like receptor signaling. Cytokine Growth Factor Rev 25:533–541. doi:10.1016/j.cytogfr.2014.06.002 25022196 PMC4254339

[26] Yamashita M, Chattopadhyay S, Fensterl V, Zhang Y, Sen GC. 2012. A TRIF-independent branch of TLR3 signaling. J Immunol 188:2825–2833. doi:10.4049/jimmunol.1103220 22323545 PMC3386560

[27] Sarkar SN, Peters KL, Elco CP, Sakamoto S, Pal S, Sen GC. 2004. Novel roles of TLR3 tyrosine phosphorylation and PI3 kinase in double-stranded RNA signaling. Nat Struct Mol Biol 11:1060–1067. doi:10.1038/nsmb847 15502848

[28] Sarkar SN, Elco CP, Peters KL, Chattopadhyay S, Sen GC. 2007. Two tyrosine residues of toll-like receptor 3 trigger different steps of NF-kappa B activation. J Biol Chem 282:3423–3427. doi:10.1074/jbc.C600226200 17178723

[29] Yamashita M, Chattopadhyay S, Fensterl V, Saikia P, Wetzel JL, Sen GC. 2012. Epidermal growth factor receptor is essential for toll-like receptor 3 signaling. Sci Signal 5:ra50. doi:10.1126/scisignal.2002581 22810896 PMC3431157

[30] Veleeparambil M, Poddar D, Abdulkhalek S, Kessler PM, Yamashita M, Chattopadhyay S, Sen GC. 2018. Constitutively bound EGFR-mediated tyrosine phosphorylation of TLR9 is required for its ability to signal. J Immunol 200:2809–2818. doi:10.4049/jimmunol.1700691 29531172 PMC5893352

[31] Sasai M, Iwasaki A. 2011. Love triangle between Unc93B1, TLR7, and TLR9 prevents fatal attraction. Immunity 35:3–5. doi:10.1016/j.immuni.2011.07.006 21777792 PMC3143494

[32] Zhu FG, Reich CF, Pisetsky DS. 2001. The role of the macrophage scavenger receptor in immune stimulation by bacterial DNA and synthetic oligonucleotides. Immunology 103:226–234. doi:10.1046/j.1365-2567.2001.01222.x 11412310 PMC1783228

[33] Killpack TL, Ballesteros M, Bunnell SC, Bedugnis A, Kobzik L, Hu LT, Petnicki-Ocwieja T. 2017. Phagocytic receptors activate Syk and Src signaling during borrelia burgdorferi phagocytosis. Infect Immun 85:e00004-17. doi:10.1128/IAI.00004-17 28717031 PMC5607427

[34] Chattopadhyay S, Veleeparambil M, Poddar D, Abdulkhalek S, Bandyopadhyay SK, Fensterl V, Sen GC. 2015. EGFR kinase activity is required for TLR4 signaling and the septic shock response. EMBO Rep 16:1535–1547. doi:10.15252/embr.201540337 26341626 PMC4641505

[35] Biswas C, Rao S, Slade K, Hyman D, Dersh D, Mantegazza AR, Zoltick PW, Marks MS, Argon Y, Behrens EM. 2018. Tyrosine 870 of TLR9 is critical for receptor maturation rather than phosphorylation-dependent ligand-induced signaling. PLoS One 13:e0200913. doi:10.1371/journal.pone.0200913 30024926 PMC6053202

[36] Wang C, Sharma N, Veleeparambil M, Kessler PM, Willard B, Sen GC. 2021. Sting-mediated interferon induction by Herpes simplex virus 1 requires the protein tyrosine kinase Syk. mBio 12:e03228-21. doi:10.1128/mbio.03228-21 34933455 PMC8689565

[37] Wang C, Wang X, Veleeparambil M, Kessler PM, Willard B, Chattopadhyay S, Sen GC. 2020. EGFR-mediated tyrosine phosphorylation of STING determines its trafficking route and cellular innate immunity functions. EMBO J 39:e104106. doi:10.15252/embj.2019104106 32926474 PMC7667877

[38] Yew KH, Carsten B, Harrison C. 2010. Scavenger receptor A1 is required for sensing HCMV by endosomal TLR-3/-9 in monocytic THP-1 cells. Mol Immunol 47:883–893. doi:10.1016/j.molimm.2009.10.009 19914718

[39] Martínez-Colón GJ, Warheit-Niemi H, Gurczynski SJ, Taylor QM, Wilke CA, Podsiad AB, Crespo J, Bhan U, Moore BB. 2019. Influenza-induced immune suppression to methicillin-resistant Staphylococcus aureus is mediated by TLR9. PLoS Pathog 15:e1007560. doi:10.1371/journal.ppat.1007560 30682165 PMC6364947

[40] Onyishi CU, Desanti GE, Wilkinson AL, Lara-Reyna S, Frickel E-M, Fejer G, Christophe OD, Bryant CE, Mukhopadhyay S, Gordon S, May RC. 2023. Toll-like receptor 4 and macrophage scavenger receptor 1 crosstalk regulates phagocytosis of a fungal pathogen. Nat Commun 14:4895. doi:10.1038/s41467-023-40635-w 37580395 PMC10425417

[41] Sanjuan MA, Rao N, Lai K-T, Gu Y, Sun S, Fuchs A, Fung-Leung W-P, Colonna M, Karlsson L. 2006. CpG-induced tyrosine phosphorylation occurs via a TLR9-independent mechanism and is required for cytokine secretion. J Cell Biol 172:1057–1068. doi:10.1083/jcb.200508058 16567503 PMC2063763

[42] Jabara HH, McDonald DR, Janssen E, Massaad MJ, Ramesh N, Borzutzky A, Rauter I, Benson H, Schneider L, Baxi S, et al.. 2012. DOCK8 functions as an adaptor that links TLR-MyD88 signaling to B cell activation. Nat Immunol 13:612–620. doi:10.1038/ni.2305 22581261 PMC3362684

[43] Khan NS, Kasperkovitz PV, Timmons AK, Mansour MK, Tam JM, Seward MW, Reedy JL, Puranam S, Feliu M, Vyas JM. 2016. Dectin-1 controls TLR9 trafficking to phagosomes containing β-1,3 glucan. The Journal of Immunology 196:2249–2261. doi:10.4049/jimmunol.1401545 26829985 PMC4761466

[44] Das S, Ghosh AK, Singh S, Saha B, Ganguly A, Das P. 2015. Unmethylated CpG motifs in the L. donovani DNA regulate TLR9-dependent delay of programmed cell death in macrophages. J Leukoc Biol 97:363–378. doi:10.1189/jlb.4A0713-378RR 25473100

[45] Wang J, Lau KY, Jung J, Ravindran P, Barrat FJ. 2014. Bruton’s tyrosine kinase regulates TLR9 but not TLR7 signaling in human plasmacytoid dendritic cells. Eur J Immunol 44:1130–1136. doi:10.1002/eji.201344030 24375473

[46] Kenny EF, Quinn SR, Doyle SL, Vink PM, van Eenennaam H, O’Neill LAJ. 2013. Bruton’s tyrosine kinase mediates the synergistic signalling between TLR9 and the B cell receptor by regulating calcium and calmodulin. PLoS One 8:e74103. doi:10.1371/journal.pone.0074103 23967355 PMC3743783

[47] Chakravarty S, Subramanian G, Popli S, Veleeparambil M, Fan S, Chakravarti R, Chattopadhyay S. 2023. Interferon-stimulated gene TDRD7 interacts with AMPK and inhibits its activation to suppress viral replication and pathogenesis. mBio 14:e0061123. doi:10.1128/mbio.00611-23 37712680 PMC10653931

[48] Chakravarty S, Varghese M, Fan S, Taylor RT, Chakravarti R, Chattopadhyay S. 2024. IRF3 inhibits inflammatory signaling pathways in macrophages to prevent viral pathogenesis. Sci Adv 10:eadn2858. doi:10.1126/sciadv.adn2858 39121222 PMC11313863

[49] Waitkus MS, Chandrasekharan UM, Willard B, Tee TL, Hsieh JK, Przybycin CG, Rini BI, Dicorleto PE. 2014. Signal integration and gene induction by a functionally distinct STAT3 phosphoform. Mol Cell Biol 34:1800–1811. doi:10.1128/MCB.00034-14 24615012 PMC4019034

